# Extinction hazards in experimental *Daphnia magna* populations: effects of genotype diversity and environmental variation

**DOI:** 10.1002/ece3.449

**Published:** 2012-12-21

**Authors:** John D Robinson, John P Wares, John M Drake

**Affiliations:** 1 Biology Department, City College of New YorkNew York, NY, USA; 2 Department of Genetics, University of GeorgiaAthens, Georgia; 3 Odum School of Ecology, University of GeorgiaAthens, Georgia; 4 Department of Zoology, Oxford UniversityOxford, UK

**Keywords:** *Daphnia magna*, environmental variation, extinction, genetic diversity

## Abstract

Extinction is ubiquitous in natural systems and the ultimate fate of all biological populations. However, the factors that contribute to population extinction are still poorly understood, particularly genetic diversity and composition. A laboratory experiment was conducted to examine the influences of environmental variation and genotype diversity on persistence in experimental *Daphnia magna* populations. Populations were initiated in two blocks with one, two, three, or six randomly selected and equally represented genotypes, fed and checked for extinction daily, and censused twice weekly over a period of 170 days. Our results show no evidence for an effect of the number of genotypes in a population on extinction hazard. Environmental variation had a strong effect on hazards in both experimental blocks, but the direction of the effect differed between blocks. In the first block, variable environments hastened extinction, while in the second block, hazards were reduced under variable food input. This occurred despite greater fluctuations in population size in variable environments in the second block of our experiment. Our results conflict with previous studies, where environmental variation consistently increased extinction risk. They are also at odds with previous studies in other systems that documented significant effects of genetic diversity on population persistence. We speculate that the lack of sexual reproduction, or the phenotypic similarity among our experimental lines, might underlie the lack of a significant effect of genotype diversity in our study.

## Introduction

Extinction is ubiquitous in natural systems and the ultimate fate of all biological populations. Indeed, the present rates of global biodiversity decline may be approaching those seen during the five mass extinction events in evolutionary history (Pimm et al. 1995; Wake and Vredenburg 2008; Barnosky et al. 2011). Anthropogenic effects such as pollution and global climate change, along with environmental stressors, are among the causes of the current elevated extinction rates (Barnosky et al. 2011). In addition, habitat fragmentation is thought to be one of the primary threats to global biodiversity this century (Hanski 1998). The result of fragmentation may be to shift once continuous populations into networks of semi-isolated habitat patches. In such metapopulations, local extinctions are common and are balanced by the recolonization of empty habitats from occupied patches (Levins 1969). Identifying the influence of population and habitat-specific characteristics on the rate of extinction is therefore an important goal in conservation biology. Natural metapopulations can contribute greatly to our understanding of population extinction, but their complexity limits our ability to dissect the influences of various factors on the risk of local extinction (Griffen and Drake 2008a).

Experimental populations in the laboratory are ideal for the study of population extinction. These systems are tractable, replicable, and obviate the need for costly and possibly unethical field manipulations or laboratory experiments using species of conservation concern (Griffen and Drake 2008a). The taxa used in these experiments often have short generation times, allowing multi-generational experiments to be conducted on the order of weeks to years. Previous studies have assessed the influences of migration, competition, habitat fragmentation, inbreeding, and a wide variety of other factors on the persistence of experimental populations (Griffen and Drake 2008a).

The impacts of biodiversity on ecosystem properties and the provision of ecosystem services are well established (Tilman et al. 1997; Loreau et al. 2001; Hooper et al. 2005; Cardinale et al. 2006). While these effects are usually considered in terms of species diversity, intraspecific genetic diversity may have similar effects on population-level processes (Hughes et al. 2008; Duffy 2009). Genetic diversity effects have primarily been observed in manipulations of genotype richness in plants (Hughes and Stachowicz 2004; Reusch et al. 2005; Crutsinger et al. 2006; Genung et al. 2010), but several examples also come from experiments in animal systems. For instance, honey bee swarms from diverse colonies founded new colonies faster and generally showed higher fitness than genetically uniform swarms (Matilla and Seeley 2007). Additionally, more diverse populations of the frog, *Rana latastei*, had higher survival rates when exposed to a novel viral pathogen (Pearman and Garner 2005).

Previous research also suggests that genetic diversity may influence the likelihood of population extinction, particularly in inbred populations (Frankham 1995). Studies that have directly manipulated the level of inbreeding in experimental populations have shown a decrease in evolutionary potential [measured as the ability of the population to adapt to stressful environments (Frankham et al. 1999)] and an increase in extinction risk at higher inbreeding coefficients (Reed et al. 2003). Furthermore, interactions between the effects of inbreeding and environmental stress may lead to greatly elevated extinction rates (Bijlsma et al. 2000; Reed et al. 2002). For instance, over the course of a fourteen-week experiment, a majority of observed extinctions in experimental populations of the mysid shrimp, *Americamysis bahia*, occurred in populations with low genetic diversity exposed to stressful environments (Markert et al. 2010). Similarly, in *Tribolium*, diverse experimental populations exhibited lower census size variation, and were subject to lower extinction risks (Agashe 2009; Agashe et al. 2011).

The *Daphnia magna* system has been used extensively as a model in experimental extinction studies. Natural populations of *D. magna* often inhabit ephemeral habitats, making individual populations subject to high extinction risks in nature (Pajunen and Pajunen 2003). Laboratory studies have manipulated various factors to assess their impact on the probability of extinction in *Daphnia*, including interspecific interactions [e.g., competition (Bengtsson 1993), competition and predation (Bengtsson and Milbrink 1995), parasite presence (Ebert et al. 2000)], levels of environmental variation (Drake and Lodge 2004), and migration rates both into a focal population (Drake et al. 2005) and between patches in a metapopulation (Griffen and Drake 2009). In spite of the number of extinction studies in *Daphnia*, to date no studies have explicitly examined the influence of genotype diversity on population extinction. Field studies in the *Daphnia* metapopulation inhabiting islands near the Tvärminne Zoological Station in Finland have documented extremely high rates of extinction in newly colonized populations (<1 year old), approaching 50% for three different *Daphnia* species (Pajunen and Pajunen 2003). Pajunen and Pajunen (2003) suggested two processes that might explain the elevated extinction rate in these young populations: rapid environmental changes after colonization and a lack of genetic diversity (assuming populations are colonized by a small number of founders). Environmental changes might lead to extinction before ephippia are produced. Such environmental influences could include changes in salinity (particularly in rock pools that are close to the shoreline) or humic matter (Pajunen and Pajunen 2003). Additionally, a lack of diversity in newly colonized pools might result in insufficient buffering against potentially rapid environmental changes (Pajunen and Pajunen 2003). Coupled with observations of inbreeding depression (Haag et al. 2002) and increasing diversity with population age (Haag et al. 2005), it seems that genetic diversity plays a role in determining the probability of persistence in the rock pool environments. Furthermore, *Daphnia* populations are known to exhibit cyclic dynamics in the field (Ebert 2005). A significant diversity effect could result if populations are buffered from these cycles through asynchronous responses of their constituent genotypes (Hooper et al. 2005), thus reducing the chance of extinction due to demographic stochasticity.

**Figure 1:**
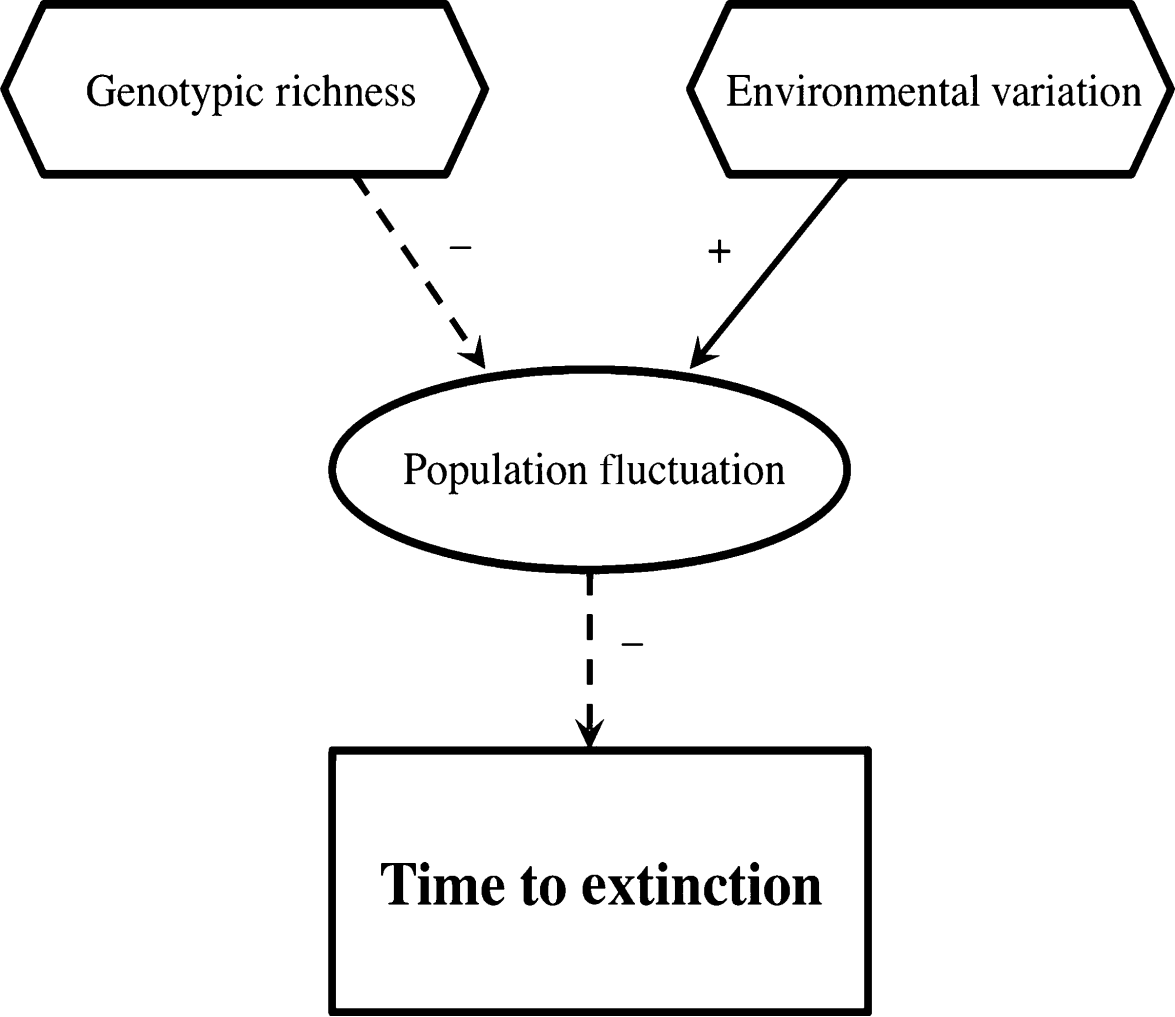
Conceptual diagram depicting the influences of our experimental treatments (environmental variation and the number of introduced genotypes) on our response (extinction time), through their hypothesized influence on population fluctuations.

The purpose of the present study was to examine the role of founding genotype richness in determining the likelihood of short-term persistence in newly colonized habitats. We included variation in food input in our experiment to determine if the effects of diversity are the same under constant and variable environments and to compare the relative influences of these factors on extinction hazards. We predicted that populations initiated with more genotypes would persist longer than monocultures under the same levels of variation in food input, either from asynchronous responses of individual genotypes (complementarity effects) or the inclusion of clones better adapted to our laboratory environment (selection effects). We also expected that environmental variation would increase the probability of extinction across our experiment overall. Finally, we hypothesized that both environmental variation and the number of genotypes would influence the magnitude of population fluctuation, and therefore the hazard of population extinction (Fig. 1). We tested these hypotheses in a pair of laboratory experiments using *Daphnia magna* lines collected and isolated from the Finnish metapopulation.

## Materials and Methods

### Collection and establishment of experimental lines

We collected *Daphnia magna* resting eggs from three islands near the Tvärminne Zoological Station in southern Finland between July 6 and July 10, 2009 [Granbusken (59.815°N, 23.246°E), Storgrundet (59.822°N, 23.261°E), and Skallotholmen (59.832°N, 23.255°E)]. Resting eggs were hatched in the laboratory under 24-hour light after a five to ten minute soak in a 5% bleach solution (Pancella and Stross 1963). *Daphnia* produce resting eggs during the sexual portion of their life cycle. For this reason, hatchlings were assumed to be genetically unique. By sampling ephippia from multiple pools and islands in the Finnish archipelago, we hoped to increase phenotypic variation and limit the influence of relatedness among lines in our experiment. This practice also makes our study less specific to the *D. magna* system; natural *Daphnia* populations are thought to be recolonized by a very small number of genotypes (Haag et al. 2005) and would be extremely unlikely to draw their founders from such a wide geographic area.

We fed *Daphnia* either live *Scenedesmus* cultured on Alga-Gro medium (Carolina Biological Supply) or a 2-mg/mL suspension of powdered *Spirulina* (JEHM Co., Inc., Trenton, NJ) in EPA hard water medium (USEPA 2002). *Daphnia* hatchlings were isolated in petri dishes for the first few days of their lives, after which they were transferred to one-quart canning jars. We propagated lines through several generations in jars, splitting the population of clones into a new jar as the density increased. After sufficient propagation, *Daphnia* lines were moved into glass aquaria (10 or 20 gallons) for stock culturing. We successfully established six *D. magna* lines in this manner. These six lines were subsequently maintained in glass aquaria and quart jars, and were used in all experiments. All aquaria, jars, and experimental containers were filled with EPA hard water medium (USEPA 2002) for *Daphnia* culture.

### Experimental design

Our experiment consisted of eight treatment combinations in which two factors were varied: the number of genotypes introduced and the level of variation in food input. Experiments were conducted in 700 mL Plexiglas containers (Griffen and Drake 2008b), into which we introduced a total of eighteen individuals, evenly distributed across one, two, three, or six randomly assigned genotypes. The practice of randomly assigning genotypes to experimental treatments led to unequal replication of individual line combinations (e.g., for two clone replicates, the 15 possible pairwise combinations were not equally represented). Also, for our highest diversity treatment (six genotypes), clones were not randomly assigned and were all included in the experimental populations and therefore do not constitute true replicates in this case (Huston 1997). Nonetheless, we assumed that the individual lines used in our experiment were exchangeable, and therefore expected that diversity effects (if present) would be detected by our approach. Daily food inputs were either constant over time [800 μL of 2-mg/mL *Spirulina* suspension daily; as in Griffen and Drake (2008b)] or were drawn from a normal distribution (mean = 800, s.d. = 1600). Randomly drawn food input values were often negative, leading to occasional days in which no food was added to a container. We kept all experimental containers under a constant 12:12 light/dark cycle at ambient room temperature (typical daily range between 22 and 26°C).

We followed a total of 160 replicate populations for 170 days. The first 80 replicates (10 for each of the treatment combinations) were founded on August 24, 2010; a second experimental block was initiated on December 1, 2010. All containers were checked daily for extinctions and census counts were recorded twice per week with the exception of the week of December 27, 2010, when a single count was made. For consistency, JR conducted all population censuses. We terminated any persisting populations after the 170-day observation period, and preserved remaining individuals in 95% ethanol.

### Characterization of lines

To characterize the life history differences among the six established lines, we followed fifteen newborn parthenotes from each line through their lives. To limit the influences of maternal effects, individuals were selected after two generations of exposure to experimental conditions. For each experimental line, fifteen “grandmothers” were isolated in 50 mL vials and sustained on 100 μL of powdered *Spirulina* suspension (2-mg/mL) per day. Offspring produced by these 90 individuals were isolated in a similar fashion until 60 “mothers” were obtained for each experimental line. The experiment to characterize the fitness of the six lines began when the 60 mothers from each line produced a total of fifteen parthenotes on the same day, for a combined total of 90 newborn individuals. On this day, all 90 experimental individuals were isolated and maintained in the same fashion as earlier generations. Each day, the number of offspring produced and a condition index for each individual was recorded. The day on which each individual died was also recorded. These data provide an independent assessment of the fitness of each of the genotypes used in the extinction experiment, and allow us to test for significant differences among our lines for several life history parameters (i.e., fecundity, condition, longevity).

### Statistical analysis

For the experiment used to characterize the fitness of our six lines, we assessed normality of the residuals of linear models relating average condition and longevity to the identity of the experimental line, using Kolmogorov-Smirnov tests. A Kruskal–Wallis rank sum test was used to test for significant differences in longevity among the six experimental lines, and an analysis of variance (ANOVA) was used to test for significant differences in average lifetime condition. Fecundity was not assessed statistically due to the very small proportion of the original 90 isolated individuals that reached sexual maturity and successfully produced offspring.

We tested for effects of the number of genotypes and the variance in food input on time to extinction and time to a population bottleneck (*n* ≤ 5 individuals) using the Cox proportional hazards model (Venables and Ripley 2010). We included the latter model to account for reductions in diversity that may have accompanied fluctuations in population size occurring well before population extinction. Additionally, because of evidence for time-varying coefficients in our dataset, we also fit a model that included the average census size from the previous week as a time-dependent covariate. The Cox model assumes an underlying hazard function, *h*_*0*_(*t*), that is altered by predictor variables through the equation:


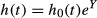
(1)

where *Y* is the combined linear effect of the predictors (i.e., *Y* = *β*_*1*_*X*_*1*_ + *β*_*2*_*X*_*2*_ + … *β*_*n*_*X*_*n*_) and *h*_*0*_(*t*) is the (unspecified) baseline hazard function (Venables and Ripley 2010). We conducted all statistical analyses in the R statistical computing environment (R Development Core Team 2012), primarily using the “survival” package (Therneau and Lumley 2009).

The predictor variables block, number of genotypes, environmental variation, and their pairwise interactions were included in a global model. We fit a Cox proportional hazards model to the data using the “coxph” function from the “survival” R package (Therneau and Lumley 2009), and tested for violations of the proportional hazards assumption using scaled Schoenfeld residuals, as calculated by the “cox.zph” function in the “survival” package (Therneau and Lumley 2009). Previous experiments have documented a two-phase extinction hazard in *Daphnia* populations, where initial population size drives early extinctions (Drake et al. 2011). Two-phase hazards result in violations of the proportional hazards assumption, and can be seen in residual plots (Drake et al. 2011). Therefore, in cases where this assumption was violated, we divided the data into subsets, based on visual inspection of the residuals, and refit the Cox model.

We also calculated two statistics that are commonly applied in biodiversity effects studies (e.g., Cardinale et al. 2006; Srivastava et al. 2009) to characterize the influence of genetic diversity on extinction time in our experiments. We included the log response ratio (*LR*), calculated as the log of the ratio of the mean extinction time in the six-clone diversity replicates to the mean extinction time in single-clone replicates. Values of *LR* less than zero indicate higher extinction risks in diverse populations (lower times to extinction), whereas positive values suggest the possibility of an ameliorating effect of genotypic richness on local extinction. Additionally, we estimated the coefficients of a power function fit to the relationship between the response variable (time to extinction, *y*) and the genotypic richness (*S*), using the equation *y = aS*^*b*^. The value of *b* from this analysis provides an indication of the size and direction of diversity effects (Srivastava et al. 2009), positive *b* would correspond to longer persistence in diverse populations.

Because of the hypothesized relationship between extinction hazard and population variability (Fig. 1), we also tested for differences in the magnitude of population fluctuation among our experimental treatments. We expected the majority of our replicate populations to exhibit population decline over the course of our experiment, therefore we used ratio-detrending to limit the influence of declining population size on our estimates of census size variability. For each replicate population, we fit a linear relationship between time and observed-census size. This relationship was then used to predict population size at the observation times, and the coefficient of variation of the ratio of the observed and predicted values was used as a measure of population variability. Subsequently, we fit a linear model to these coefficients of variation that included the predictors environmental variation and number of genotypes. Because of deviations from normality in the residuals, we used Krusall–Wallis rank sum tests (Sokal and Rohlf 1995) to test for significant differences in census size variability among experimental treatments (number of genotypes/environmental variation).

## Results

### Fitness of lines

The experiment to characterize the fitness of the six experimental lines lasted a total of 22 days, with individual longevities ranging from one day to 22 days. A Kolmogorov-Smirnov test strongly rejected (*P* ≪ 0.001) normality of the residuals of the linear model considering differences in longevity among clones, but a similar test did not reject normality of residuals for the model of average lifetime condition (*P* = 0.07). For this reason, we used a nonparametric Kruskall–Wallis rank sum test to compare longevity and an ANOVA to test for differences in average lifetime condition. No significant differences were detected among lines for longevity (*χ*^2^ = 8.6058, d.f. = 5, *P* = 0.1259) or the average lifetime condition (*F* = 1.961, d.f. = 5, *P* = 0.0941). Over the course of the experiment, only seven of the 90 individuals (from three of the six lines) successfully reproduced parthenogenetically. Because of the failure of several lines to produce offspring, and the small number of clutches for lines that did reproduce, we did not test for significant differences in fecundity among lines.

### Testing the proportional hazards assumption

**Figure 2:**
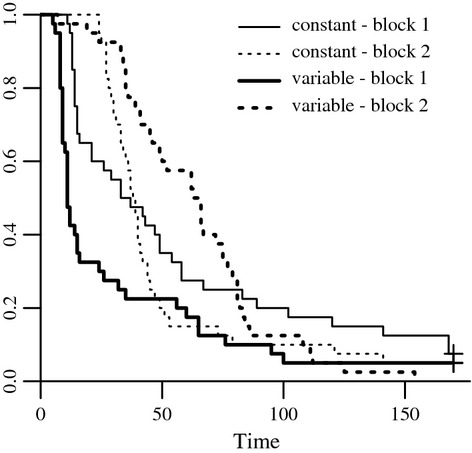
Observed survivorship curves for the two environmental variation treatments in each of the two blocks of the experiment. Solid lines are for data from block 1, while dotted lines indicate curves for block 2. Heavy lines correspond to survivor curves in the variable environments and lighter lines represent constant environments.

We observed a total of 153 extinctions from the 160 experimental populations (75 from the first block and 78 from the second). The earliest extinctions happened in less than a week (minimum time to extinction = 5 days) while the latest happened after 168 days. Extinction events were clustered in the early days of the experiment for both environmental variation treatments in the first block, with 41 of the 80 experimental populations extinct by day 20. By contrast, in the second block persistence times were generally longer, with only two populations extinct over the same period of time. Additionally, in the second block, replicates exposed to variable environments generally showed longer persistence than constant environment replicates (Fig. 2). In the first block of the experiment, 26 of the 38 observed variable environment extinctions occurred in the first 15 days. In comparison, only three variable environment populations were extinct by day 30 in the second block of our experiment.

**Figure 3:**
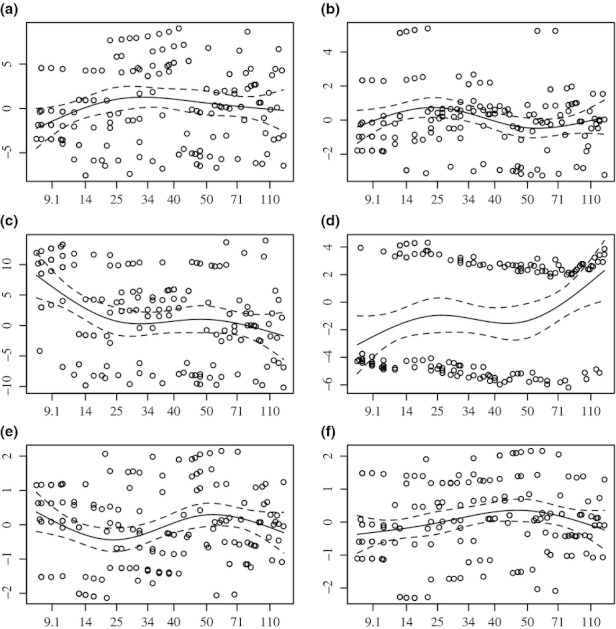
Plots of the scaled Schoenfeld residuals versus time for the Cox proportional hazards model fit to the entire dataset (both blocks). The solid line (*β*_*t*_) gives the estimated effect of the predictors through time in the experiment (with confidence intervals). Plots are given for the predictors: a) block, b) number of genotypes, c) environmental variation, d) block x environment, e) block x genotypes, and f) genotypes x environment. Significant violations of the assumption of proportional hazards were found for the predictors: block (a) and environmental variation (c).

The Cox proportional hazards model, when fit to the full dataset, violated the assumption of proportional hazards. Specifically, we found significant deviations for both environmental variation and the interaction between block and environmental variation (Table 1a). Investigation of the scaled Schoenfeld residuals (Fig. 3) suggested that a two-phase extinction hazard might underlie these deviations (Drake et al. 2011). As noted above, the majority of early extinctions occurred in the first experimental block. For this reason, we analyzed the two blocks separately. Violations of the proportional hazards assumption were evident in the first, but not the second experimental block. Data from the first block were divided into two subsets at day 20. We then refit the Cox proportional hazards model and found no violation of the proportional hazards assumption in the three subsets: early extinctions from block 1, late extinctions from block 1, and all replicates from block 2. For the model considering the time to a population bottleneck, no significant deviations from the proportional hazards assumption were detected (Table 1b). When census size was included as a time-dependent covariate, the predictors environmental variation, census, the block × census interaction, and the interaction between environmental variation and block were all found to vary with time, violating the assumption of constant hazard rates (Table 1c).

**Table 1:** Results of tests for violation of the assumption of proportional hazards

Model	Parameter	Chi-squared	*P*-value
A	Block	0.882	0.348
	Genotypes	1.648	0.199
	Environmental Variation	11.456	0.0007***
	Block × Environment	8.657	0.003**
	Block × Genotypes	0.585	0.444
	Genotypes × Environment	2.229	0.135
B	Block	3.378	0.066
	Genotypes	0.957	0.328
	Environmental Variation	0.684	0.408
	Block × Environment	0.224	0.636
	Block × Genotypes	0.409	0.523
	Genotypes × Environment	1.431	0.232
C	Block	5.552	0.019*
	Genotypes	1.139	0.286
	Environment	11.237	0.0008***
	Census	0.260	0.610
	Block × Environment	5.334	0.021*
	Block × Genotypes	0.144	0.705
	Block × Census	4.466	0.035*
	Genotypes × Environment	1.170	0.279
	Genotypes × Census	0.619	0.431
	Environment × Census	2.323	0.127

Results given are for the Cox proportional hazards model fit to a) the full extinction model (both blocks), b) the full bottleneck model, and c) the global model including a time-dependent covariate (census size). Chi-squared values and their associated significance *(P-*value) are given.

* *P* < 0.05.

** *P* < 0.01.

*** *P* < 0.001.

### Modeling extinction

For populations in block 1 that went extinct before day 20, the only significant predictor of extinction hazard was the level of environmental variation. Populations exposed to variable environments were subject to an extinction hazard that was more than 7.5 times greater than that under constant food input (Table 2a). In contrast, the influences of the number of genotypes and the interaction between genotypes and the experimental environment were not significantly different from zero. Estimated coefficients for the three predictor variables were similar in the second portion of block one and in the full dataset from the second block (Table 2a). However, the only significant effect was for the coefficient associated with environmental variation in the second block, where populations exposed to variable food inputs were at a substantially lower extinction hazard (∼35% of the hazard in constant environments; Table 2a).

**Table 2:** Estimated coefficients for predictors of the fitted models: a) two-phase extinction model, b) bottleneck model, c) model including census size as a time-dependent covariate

Model	Predictor	Estimate	Exp(est.) [95% C.I]	*P*-value
A-1.1	Genotypes	0.0223	1.023 [0.790–1.323]	0.870
	Environment	2.0351	7.653 [1.872–31.287]	0.005**
	Genotypes × Environment	−0.3061	0.736 [0.523–1.038]	0.080
A-1.2	Genotypes	−0.0649	0.937 [0.719–1.222]	0.632
	Environment	−0.3498	0.705 [0.189–2.636]	0.602
	Genotypes × Environment	0.1184	1.126 [0.752–1.684]	0.565
A-2	Genotypes	−0.0663	0.936 [0.778–1.126]	0.482
	Environment	−1.0427	0.353 [0.144–0.861]	0.022*
	Genotypes × Environment	0.1996	1.221 [0.944–1.580]	0.129
B	Block	0.4630	1.589 [0.792–3.187]	0.192
	Genotypes	0.2068	1.230 [0.917–1.650]	0.168
	Environment	1.8655	6.459 [2.068–20.170]	0.001**
	Block × Environment	−1.2717	0.280 [0.148–0.533]	0.0001***
	Block × Genotypes	−0.1497	0.861 [0.720–1.030]	0.100
	Genotypes × Environment	0.1726	1.188 [0.993–1.422]	0.059
C	Block	−0.5996	0.549 [0.215–1.403]	0.210
	Genotypes	0.1021	1.108 [0.821–1.493]	0.503
	Environment	1.4378	4.211 [1.305–13.589]	0.016*
	Census	−0.0854	0.918 [0.885–0.952]	0.000004***
	Block × Environment	−0.8575	0.424 [0.208–0.867]	0.019*
	Block × Genotypes	−0.0837	0.920 [0.757–1.117]	0.399
	Block × Census	0.0369	1.038 [1.017–1.059]	0.0004***
	Genotypes × Environment	0.0158	1.016 [0.852–1.212]	0.861
	Genotypes × Census	0.0021	1.002 [0.998–1.007]	0.344
	Environment × Census	−0.0031	0.997 [0.976–1.018]	0.773

Model 1.1 was fit to data for the first twenty days of the first block, model 1.2 considered the remaining 150 days of the first block, and model 2 included the entire dataset for the second experimental block. Coefficients are given along with the exponentiated estimate (including 95% confidence intervals), and *P*-values assessing the significance of each predictor. The exponentiated coefficient gives the proportional change in hazard for a unit increase in the predictor.

* *P* < 0.05.

** *P* < 0.01.

*** *P* < 0.001.

### Modeling population bottlenecks

Similar to our practice for the model of extinction hazard, we fit a global model including all three predictor variables (block, environmental variation, number of genotypes) and their pairwise interactions. We estimated parameter values and confidence intervals for the six parameters of the global model. Of these, only the coefficients for environmental variation and the interaction between environmental variation and block were significantly different from zero (Table 2b). Point estimates for the model coefficients suggest that in the first block of the experiment, populations exposed to variable environments experienced extinction hazards that were 81% higher than in constant environments [hazard ratio = (*e*^1.8655−1.2717^)/(1) = 1.81]. Similar to the results for extinction hazard, this trend was reversed in the second block. There, populations exposed to variable environments were at lower hazards than constant environment replicates [hazard ratio = (*e*^1.8655+2*−1.2717^)/(1) = 0.51].

### Including a time-dependent covariate

To attempt to account for the time-dependence in our global model on the full dataset, we also fit a model that included the time-dependent effects of census size on extinction hazard. Despite violations of the assumptions of the Cox model for several predictors (Table 1c), we fit the global model and focused our analysis on significant predictors of extinction hazard. Only the predictors census, block, block*environmental variation, and block*census were found to be significantly different from zero. As in both previous models, estimated coefficients (Table 2c) suggest that environmental variation had a strong positive influence on extinction hazard in the first block (variable environment populations were subject to an extinction hazard that was ∼79% higher than in constant environments), and this effect was reversed in the second block (variable environment hazards were only 76% of those in constant environments). Increases in census size led to substantial reductions in the hazard rate, with a larger effect in the first experimental block. There, a population of 40 individuals had an estimated hazard rate that was approximately 62% lower than that in a population half the size. In the second block, similar increases led to hazards that were 21% lower in the larger population. (Table 2c)

### Measuring diversity effects

We calculated two statistics that are commonly reported in biodiversity experiments (*e.g*. Cardinale et al. 2006; Srivastava et al. 2009): the log response ratio, *LR*, and the exponent of the power function*, b*. The *LR* between six-clone and single-clone replicates suggests a negative effect of genotypic richness on population persistence in both constant (*LR* = −0.4) and variable (*LR* = −0.418) environments. In contrast, the exponent of the power function shows a positive relationship between genotypic richness and the time to population extinction in both constant (*b* = 2.068) and variable (*b* = 2.059) environments, suggesting that diversity increased persistence time in our experiment.

### Effects on population fluctuations

**Figure 4:**
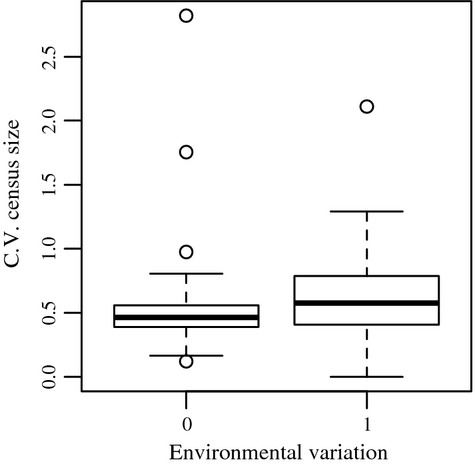
Boxplots of the ratio-detrended coefficients of variation in census size over the course of the experiment in constant (environmental variation = 0) and variable (environmental variation = 1) environments. The heavy line in the center of each box gives the location of the median, while the upper and lower faces of the box plot the 25th and 75th percentiles. The whiskers extend to the range, excluding outliers (plotted as open circles).

We examined ratio-detrended coefficients of variation in population size within replicates, calculated from biweekly census counts over the course of our experiment. These data support the hypothesis that populations in variable environments would exhibit significantly higher coefficients of variation in census size (*χ*^2^ = 8.734, d.f. = 1, *P* < 0.01; Fig. 4). When the two experimental blocks were considered separately, this pattern was present in the second (*χ*^2^ = 26.4033, d.f. = 1, *P* ≪ 0.001), but not the first (*χ*^2^ = 0.4537, d.f. = 1, *P* = 0.5006) block. Overall, census size variation was not significantly different between the two blocks (*χ*^2^ = 1.9961, d.f. = 1, *P* = 0.1577). Furthermore, we found no evidence for differences in census size variation among populations with differing numbers of genotypes (*χ*^2^ = 0.7454, d.f. = 3, *P* = 0.8625).

## Discussion

Our experiment was designed with two primary questions in mind. First, does the number of *D. magna* genotypes that colonize a population affect the time to extinction (in constant and/or variable environments)? Second, does environmental variation reduce the time to extinction? Results of our experiment stand in sharp contrast with our expectations and hypotheses. We expected that genetic diversity would facilitate population persistence, and that populations exposed to variable environments would be subject to consistently higher extinction hazards. When fit to the raw data from our experiment, the exponent of the power function (*b* ∼ 2) is greater than 0, indicating a positive effect of diversity on time to extinction. However, the log response ratios were negative in both environments (*LR* ∼ −0.4), with the highest diversity treatment having shorter average persistence times than monocultures. Our Cox models found no evidence for a significant effect (positive or negative) of the number of introduced genotypes on population persistence. In contrast to our expectations, environmental variation had a variable effect, increasing hazards in the first experimental block and decreasing them in the second. We also expected that diverse populations would show less variation in census size, and that diversity would buffer greater variation in abundance in variable environments. There was no evidence for a buffering effect of diversity in our experiment, but populations exposed to variable food input did tend to be more variable themselves. It is possible that experimental conditions differed between the two blocks of our experiment, but it seems highly unlikely that they would differ enough to lead to a complete reversal in the patterns of extinction in variable environments. Therefore, we focus the discussion on potential biological causes for the lack of a significant diversity effect in our experiment.

Previous studies have documented the effects of genetic diversity on a variety of population processes (see review in Hughes et al. 2008). More specifically, extinction experiments have generally supported the notion that diverse populations are more likely to persist under stressful or novel conditions (Frankham et al. 1999; Reed et al. 2002; Markert et al. 2010). Nonetheless, our analyses show no effect of the number of introduced genotypes on persistence in populations of *D. magna*. Most previous studies include sexual reproduction in the experimental populations, but over the 170-day experimental period, reproduction in our laboratory populations was exclusively clonal (i.e., parthenogenesis). We speculate that the effects of diversity on persistence and adaptation may be more pronounced in cases when inbreeding depression is a possibility in the population. Additionally, if one of our clonal lines had been better adapted to the laboratory environment, we would expect to have detected a significant effect of diversity on population persistence. An alternative possibility is that the similarity among the experimental lines, in terms of average lifetime condition and longevity, limited the possibility for selection effects in our experiment.

In the first experimental block, as expected, populations maintained on variable food inputs were subject to higher extinction hazards. However, the reverse was true in the second experimental block, despite greater fluctuations in census size in populations exposed to variable environments. Generally, persistence was much longer for both treatments in the second experimental block. This pattern might be the result of differences in the quality of the food source between the two blocks, which might also have contributed to the observed violation of model assumptions. Alternatively, the greater length of exposure to the laboratory environment, and in particular, the *Spirulina* food source in *Daphnia* stock aquaria may have allowed the lines to adapt to our experimental conditions before initiation of the second block of the experiment (100 days after the start of the first block). *Daphnia* are known to exhibit plastic responses to algal composition (Hairston et al. 2001; Ghadouani and Pinel-Alloul 2002; Bednarska and Dawidowicz 2007), including changes in the morphology of the filtering apparatus. It is therefore possible that phenotypic plasticity in response to a suboptimal food source (powdered *Spirulina*) led to longer persistence times in the second experimental block.

Previously, increased food variation, at levels comparable to those in our experiment, decreased persistence times in experimental populations of *D. magna* (Drake and Lodge 2004). We expected variation in resource availability (food input) to increase the variability in population size and therefore, consistently increase the risk of extinction (Griffen and Drake 2008a). Coefficients of variation confirmed the prediction that populations exposed to variable environments would exhibit greater variation in census sizes (Fig. 4), at least in the second block of our experiment. In contrast with studies in *Tribolium* (Agashe 2009), we failed to detect differences in the CV of census size among our diversity treatments.

In summary, our experiment was designed to test for influences of environmental variation and founding genotype diversity on the time to extinction in *Daphnia* populations. Interestingly, levels of population fluctuation were elevated in replicates exposed to variable environments during the second block of our experiment, but not during the first block. This increased census size variation did not appear to increase the risk of extinction, as environmental variation in the second block appeared to have a negative effect on the hazard of population extinctions or reductions and persistence times were generally longer in the second experimental block. The positive effect of environmental variation on population persistence in the second block of our experiment is at odds with previous studies manipulating food input in the *D. magna* model system (Drake and Lodge 2004). Our results concerning the effects of genetic diversity are unequivocal. In the absence of sexual reproduction, our data show no evidence of a mediating effect of the number of genotypes on extinction hazards or the degree of population fluctuations. Future studies that seek to assess the potential for effects of genetic diversity in *D. magna* should select genotypes with measurable differences in fitness-related traits and include the sexual portion of the *Daphnia* life cycle in their experiments to allow for inbreeding depression in low diversity populations.
